# Smartphone-based application to control and prevent overweight and obesity in children: design and evaluation

**DOI:** 10.1186/s12911-023-02304-2

**Published:** 2023-10-04

**Authors:** Zahra Zare, Elmira Hajizadeh, Maryam Mahmoodi, Reza Nazari, Leila Shahmoradi, Sorayya Rezayi

**Affiliations:** 1 Department of Operating Room, School of Allied Medical Sciences, Tehran University of Medical Sciences, Tehran, Iran; 2 Department of Health Information Management and Medical Informatics, School of Allied Medical Sciences, Tehran University of Medical Sciences , Tehran, Iran; 3 Department of Cellular and Molecular Nutrition, School of Nutritional Sciences and Dietetics, Tehran University of Medical Sciences, Tehran, Iran

**Keywords:** Application, Usability evaluation, Obesity, Overweight, Smartphone

## Abstract

**Background:**

Obesity is a multifaceted condition that impacts individuals across various age, racial, and socioeconomic demographics, hence rendering them susceptible to a range of health complications and an increased risk of premature mortality. The frequency of obesity among adolescent females in Iran has exhibited an increase from 6 to 9%, while among boys, it has risen from 2 to 7%. Due to the increasing prevalence and advancements in technology, the primary objective of this study was to develop and evaluate a smartphone-based app that would serve as an educational tool for parents about the matter of childhood overweight and obesity. Additionally, the app aimed to enhance parents’ capacity to effectively address and manage their children’s weight-related concerns.

**Methods:**

The design of the present study is of an applied-developmental type. In the first phase, the content of related smartphone-based app was determined based on the needs identified in similar studies and the findings of a researcher-made questionnaire. The versions of the app were designed in the android studio 3 programming environment, using the Java 8 programming language and SQLite database. Then, in order to evaluate the app’s usability, ease of access, and different features, the standard usability evaluation questionnaire and the user satisfaction questionnaire (QUIS) were completed by the users.

**Results:**

The developed app has five main sections: the main page, recommendation section (with eight parts), charts over the time, child psychology, and reminders for each user. The designed app was given to 20 people including nutritionists and parents with children under 18 years of age for conducting usability evaluation. According to the scores of participants about the usability evaluation of the app, it can be concluded that groups participating in the study could use the program, and they rated the app at a “good” level. Overall performance of the app, screen capabilities, terms and information of the program, learnability, and general features are scored higher than 7.5 out of 9.

**Conclusion:**

By using this app, people can become familiar with the causes and symptoms of weight imbalance and manage their weight as best as possible. This app can be considered as a model for designing and creating similar broader systems and programs for the prevention, management, treatment and care of diseases, which aim to help control diseases as much as possible and increase the quality of life and reduce complications for be patients.

## Background

Most of the world’s population live in countries where overweight and obesity cause more deaths than underweight and in fact, obesity and overweight are among the main causes of disability and premature death in the world [1]. Obesity is the biggest and most important health problem in the world due to its high prevalence in societies, the high costs it imposes on healthcare systems, and also the complications and problems it causes to people [2]. During the last three decades, the prevalence of obesity in children and adolescents worldwide has increased rapidly [3, 4]; so that among children aged 6–11, it has increased from 5.6 to 17% [3]. The information obtained from domestic studies also indicates a high prevalence of obesity in Iran and makes clear the need to take preventive measures and identify high-risk groups [5]. While the incidence of obesity and related mortality is higher in Asian countries than in other countries, the high prevalence of obesity is more commonly recorded in Eastern European and North American countries than in other areas. Significantly, there has been a notable growth in the prevalence of obesity among adolescent females in Iran, with an increase from 6 to 9%. Similarly, among adolescent males, the incidence of obesity has experienced an upward trend, escalating from 2 to 7%. There is an urgent need for preventative interventions to address the dual burden of malnutrition and obesity, together with the modifiable environmental variables linked with both conditions, among Iranian children and the general population [6–8].

Notably, there is a close relationship between obesity in childhood and the increased possibility of obesity in adulthood as well as the resulting physical problems. This relationship makes obesity a public health concern for children and adolescents [9]. In today’s world, obesity in children leads to a lower quality of life in adults, causing them to spend a higher level of medical expenses in the future [10]. It also has a significant impact on the mental and emotional health of children, adolescents, and adults [11]. It is important to note that obesity in infancy, childhood, and adolescence has adverse consequences and can negatively affect almost every system in the body. Obesity is an important risk factor for complications, such as high blood pressure, diabetes, insulin resistance, dyslipidemia, polycystic ovary syndrome, liver dysfunction, kidney failure, tooth decay, orthopedic/mobility problems, and respiratory problems including sleep breathing disorder and obstructive sleep apnea [12].

Therefore, with increasing obesity rates and related health problems, finding ways to help people lose excess weight is crucial, because overweight and obesity in children are associated with many adverse physical and psychological consequences [13]. This is despite the fact that overweight and obesity can be identified, prevented, and treated, and its early diagnosis can lead to timely intervention [14]. In this regard, Information and Communication Technology (ICT) can play a major role by providing easier and more affordable access to health and treatment services [15]. Using communication technology to provide health services and manage them properly by modifying people’s behaviour and encouraging self-care might involve a developing trend in medical education, treatment, and prevention. In the process of changing self-care behavior, patients should be encouraged to take responsibility for their own care [16]. An empowerment program focusing on awareness, knowledge, and motivation leads to self-efficacy promotion and finally improvement of self-care behaviors [17]. With the increase in people’s interest in this communication tool, an opportunity has been created for health experts to educate people and increase the health of society [18]. Also, smartphone services can overcome time and place limitations and make health care more accessible, especially for people who live in remote areas [19]. This technology also provides a suitable ground for fair delivery of health services to everyone through the entire care chain [20, 21].

In addition, smartphone-based applications, while making it easier to establish a close relationship with patients, will minimize the need for face-to-face interventions and as a result, reduce the costs and increase the effectiveness of health care services [22]. The advantages of such technologies are more evident, especially in epidemic/pandemic conditions such as COVID-19, which highlighted the need to reorganize and prioritize alternative health care methods in patient management and treatment while providing health systems with a key solution to such challenges [23]. Studies have shown that in many countries, telephone services are known as the most efficient and cost-effective method in the follow-up of chronic diseases because it reduces the patient’s need for unnecessary hospital visits [24]. In recent years, the use of smartphone-based applications for monitoring and self-care has become popular, and many articles on the development of these applications are presented [25]. In 2021, an app has been designed to monitor the condition of patients with urinary tract stones [26]. Also, in 2022, an app has been developed to predict the survival of the transplanted kidney [27]. According to the Technology Acceptance Model (TAM), a mobile app for monitoring patients with Alzheimer’s disease is designed and evaluated [28]. A separate initiative is developing a mobile app to assist service users in receiving safety planning and mental health support [29].

Since health education, as an educational process, tries to change lifestyles by combining different perspectives, and also helps individuals, families, and communities to make informed decisions about their health-related issues, it can play an important role in institutionalizing preventive behaviors in children and adolescents in regard to obesity and overweight [30].

### Significance of this work

The aim of the present study was to design and evaluate a smartphone-based app to educate parents about the issue of overweight and obesity in children and increase their ability to manage their children’s weight. Studies carried out in our nation indicate that no application programme has been created to use mobile phone technology in the area of overweight and obesity prevention and control, particularly for children in the nation. As a result, this research creates educational axes in sports, nutrition, and other areas in order to augment previous research’s experiences in controlling weight and eradicating harmful habits. It is anticipated that parents who use this software will play a more helpful and proactive role in ensuring that their children eat a nutritious diet and maintain a healthy weight. It can be argued that this app can detect, prevent, and treat obesity and overweight, and that early detection allows for prompt intervention in youngsters.

## Materials and methods

The present study is of the applied-developmental type and has been carried out in two separate phases, including the first phase of preliminary design and usability evaluation and the second phase of the final design based on the corrective comments of the evaluators.

Determining the content of a smartphone-based app to control and prevent overweight and obesity in children is performed in the previous work of the authors. So, we have previous work methods [31] and present work methods.

### Previous work

This app is designed based on literature review and survey of experts. The content of this app was determined based on the needs identified from similar studies and the findings of a researcher-made questionnaire. This questionnaire had four parts, including demographic data, evaluation data, treatment recommendations, and practical capabilities, which were approved by 30 nutritionists. The exact details of the app content and its information elements have been published in the authors’ previous research [31].

### Present work

The principal two phases of this research are presented in the following:

#### The first phase

The first phase contains the initial design and usability evaluation of a smartphone-based app to control and prevent overweight and obesity in children.

At this stage, based on the results of the previous paper as well as the feedback and comments received from the users, the initial design of the app was carried out, and the designed app was given to 20 people (including nutritionists and parents with children under 18 years of age) to use and review it. The selection criteria for the samples were availability, having a phone with Android operating system version 3 and above, being familiar with mobile applications, and being interested in participating in the research evaluation. They were asked to evaluate the designed app for 10 days and use its capabilities in order to identify any potential bugs and issues related to its design and user interface. In this regard, the necessary explanations were given to the selected users on how to enter information and use its features. The initial version was designed in the Android Studio 3 programming environment, using the Java 8 programming language and the SQLite database.

Then, in order to evalaute the application’s usability, ease of access, and different features, the standard usability evaluation questionnaire and the user satisfaction questionnaire (QUIS) were completed by the users. The validity and reliability of the QUIS questionnaire has been confirmed in the former studies [32, 33].

QUIS has 30 questions on a 9-option Likert scale. The first part of the questionnaire includes three questions about the sample’s personal information, and the rest are related to the overall function of the system, its screen capabilities, its use of terms, the system’s learning capabilities by users, and the general capabilities of the system. For each question, an answer with a score of zero (lowest) to nine (highest) is considered. To analyze the data, the scores are classified into three levels: scores of 0 to 3 indicate a poor level, scores of 3.1 to 6 indicate a moderate level, and scores of 6.1 to 9 indicate a good level. Data obtained in the first phase of the research were analyzed by SPSS software version 22, using descriptive statistics such as mean and percentage.

#### The second phase

The second phase includes the final design of a smartphone-based app to control and prevent overweight and obesity in children.

In response to the users’ suggestions in the first part of the second phase, more educational information related to supplementary nutrition and nutritional diets was added to the app. Some users wanted the evaluation results to be simplified and explained as much as possible in terms of growth tables, and others wanted the font and color of some pages to be changed. Therefore, after making the corrections suggested by the users, the final version of the app was created. It should be noted that in this app, the existence of charts and instructions on how to use them will provide children and their parents with appropriate feedback on the weight management process, which can be a suitable and important incentive for them. This app can be installed on smartphones with the Android operating system.

## Results

After determining the information elements in four areas of demographic data, evaluation data, treatment recommendations, and functional capabilities in the first publication of authors, in this research, we designed and evaluated a smartphone-based app to control and prevent overweight and obesity in children.

The Minimum Data Set (MDS) required for designing the app for overweight and obesity management in children and adolescents was modified in our previously published paper [31] based on the data from the guidelines of the United States, Canada, Australia, Britain, Iran, and experts’ opinions. The importance of this MDS suggested was calculated based on the percentage points given by experts for the demographic data of 100%, the assessment data of 88.33%, the therapeutic recommendations of 97.67%, and the app capabilities of 88.94%.

### User interface of the app

#### Main pages

**Fig. 1:**
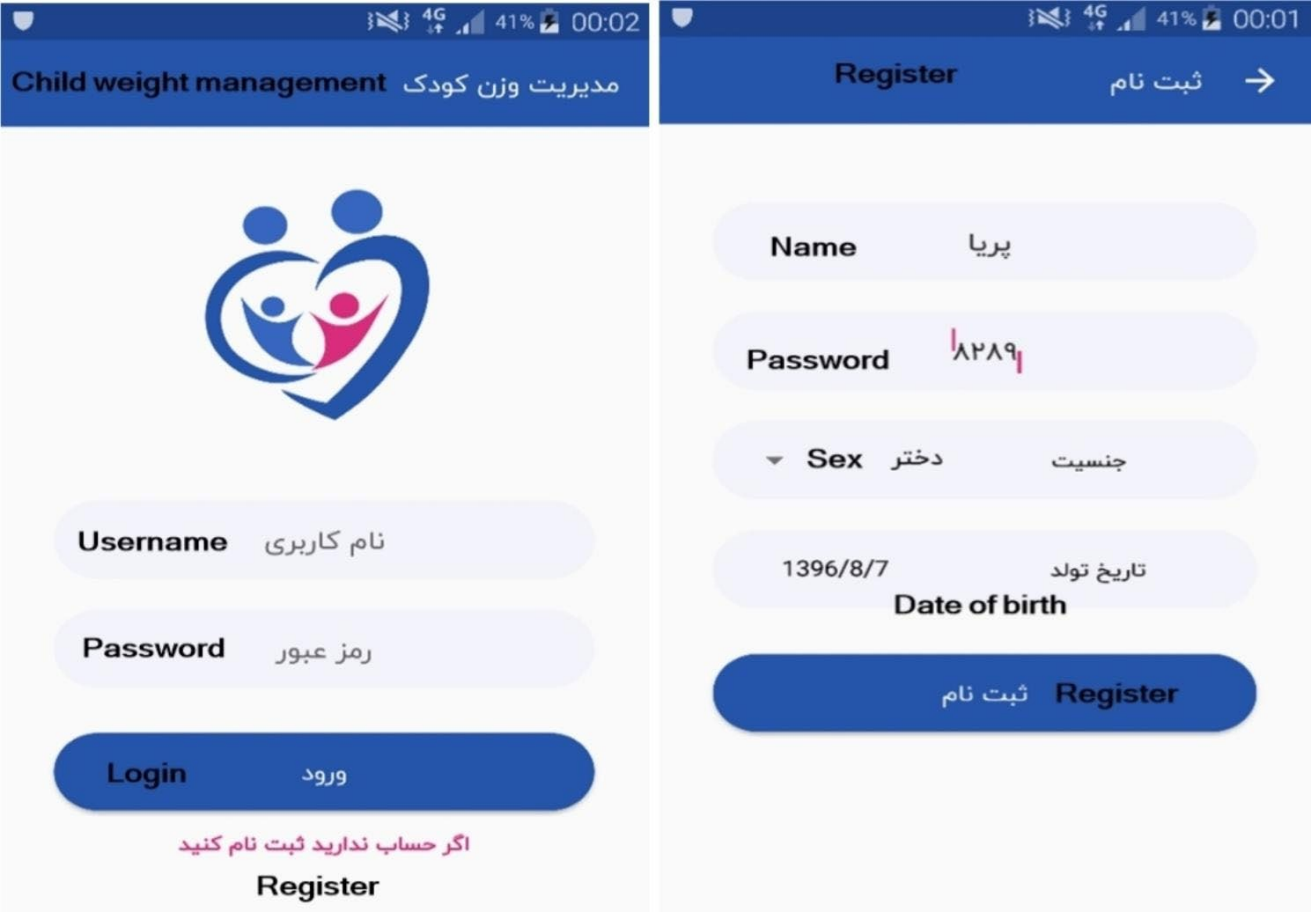
App login and registration

Below is a summary of the application’s content and capabilities. Each page of the app has been described in detail:

The first page is related to the “program login and logo”. The second page is related to “registration”, where the user enters his/her demographic information (including username, password, gender, and date of birth) to complete the registration process (Fig. 1).

The next page is the “home page” which contains the main menu of the program, which includes a growth measurement section or registration of growth information, general recommendations, trend charts, psychological assessments of the child, and reminders. Some images of these pages are provided below. Information such as weight, height, and date of measurements is included in the “growth measurement section or registration of growth information”, which is completed and saved by the user. Also, there is an icon for “correct height and weight measurement” on that page, which provides relevant explanations to users.

**Fig. 2:**
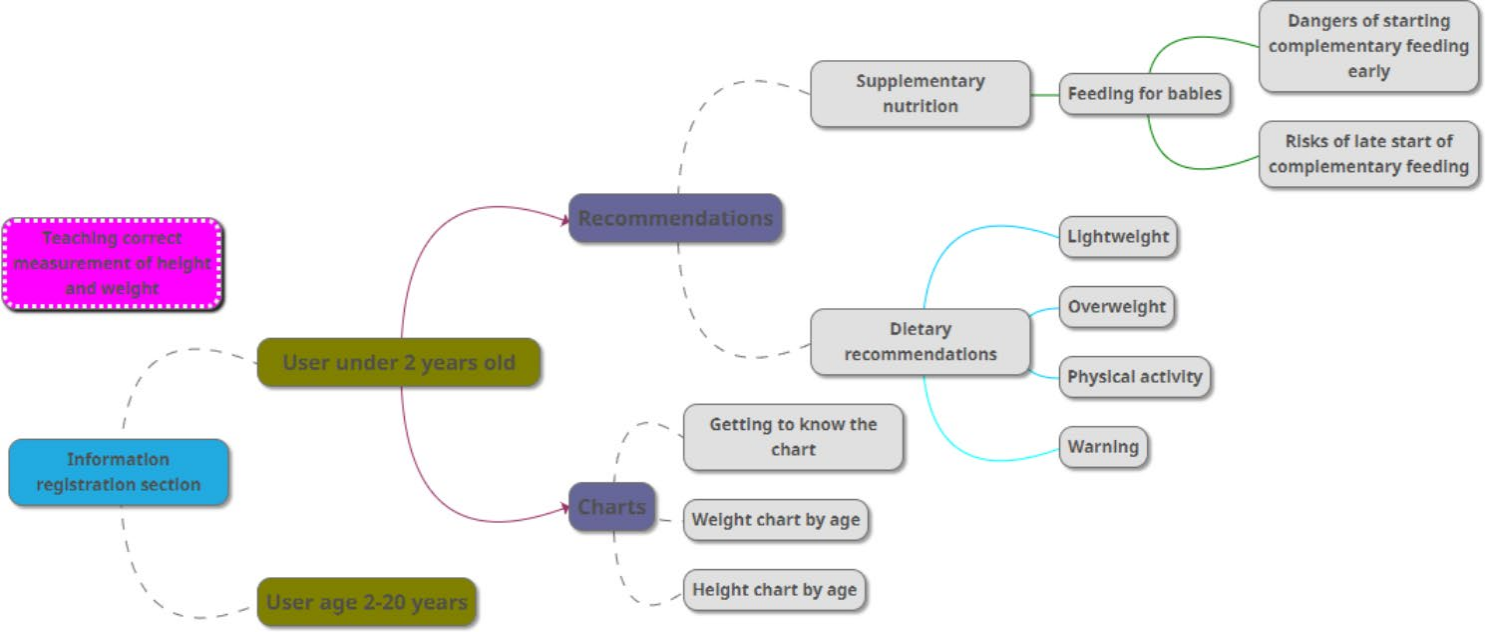
The application’s content for a user under the age of 2

**Fig. 3:**
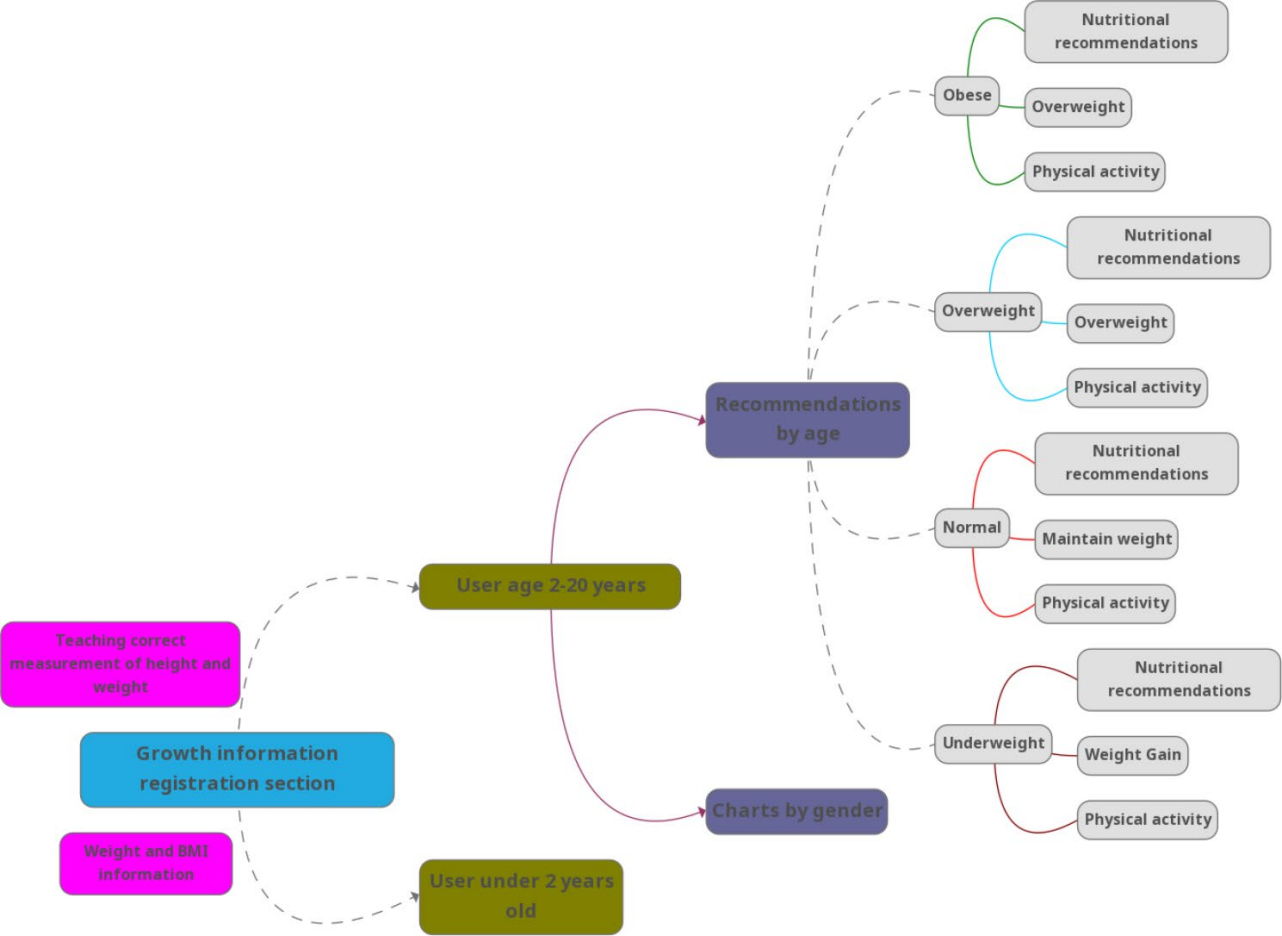
The application’s content for a user between the age of 2 and 20 years

**Fig. 4:**
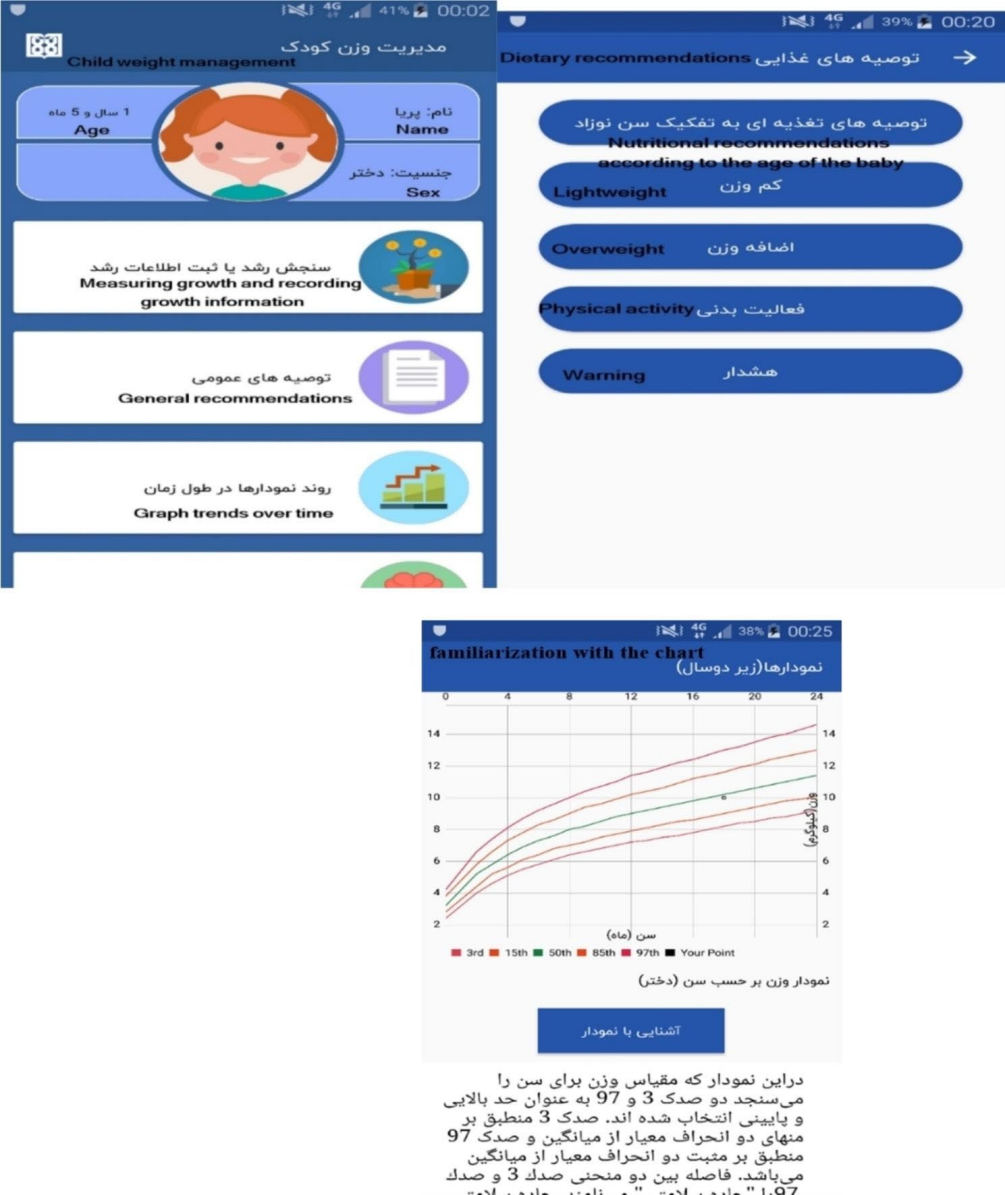
Pages for under two-year-old child

After entering the user’s information on the growth measurement page and clicking on the “calculate” option, a page will open that differs depending on the age group of the user. In fact, this program has the ability to act intelligently in providing educational information as well as diagrams and charts necessary to interpret the child’s development. Also, according to the child’s age group, it can provide completely different recommendations, training, and charts. The charts are also presented differently and separately according to the user’s gender because all these charts are different for boys and girls and presenting them all at once increases the possibility of error in choosing the correct chart by parents. These differences are presented in Figs. 2 and 3. Three different pages for children under two years old are provided in Fig. 4. On these pages, for users under the age of two years, there is an option called “familiarization with the chart” that briefly describes the charts (which have been provided by WHO) and teaches parents how to use them. The app also warns parents when to be worried about their children’s health or refers them to the relevant specialist.

**Fig. 5:**
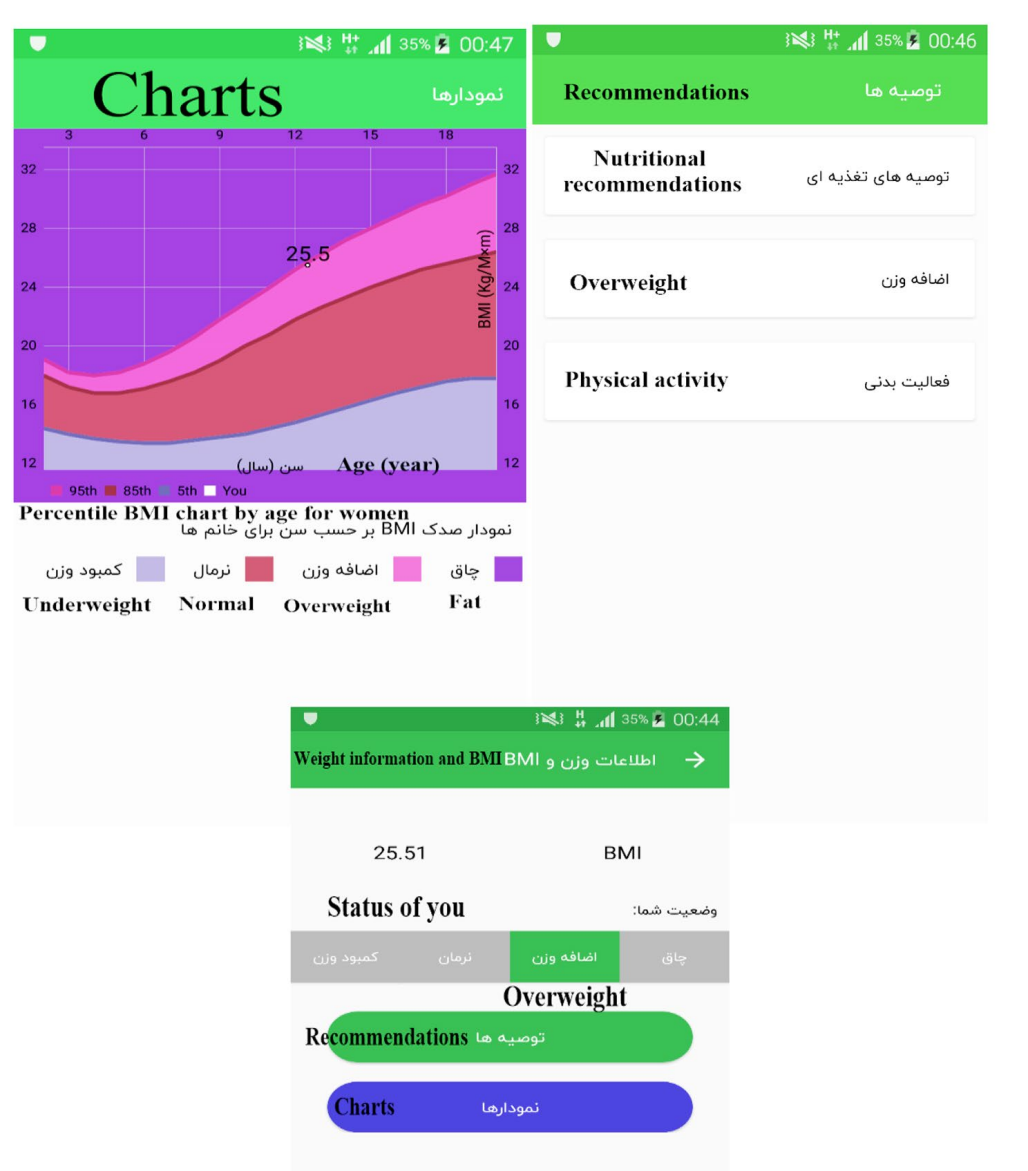
Example of recommendations and diagrams related to the age group of 11–20 years

If the users are in the age group of 2–20 years, after receiving information on the growth measurement and calculation, they can enter a page called “weight and BMI information”, where their BMI values are presented on a spectrum that ranges from obese to underweight (Fig. 5). At the bottom of this spectrum, two main options of “recommendations” and “diagrams” can be seen. These recommendations are suitable for three different age groups: 3–5, 5–10, and 11–20 years. In fact, it should be said that the program has the intelligence to provide users with specific recommendations based on their age group and gender. The following is an example of one of these recommendations and diagrams, which is related to the age group of 11–20 years (Fig. 5).

### The section of general recommendations

**Fig. 6:**
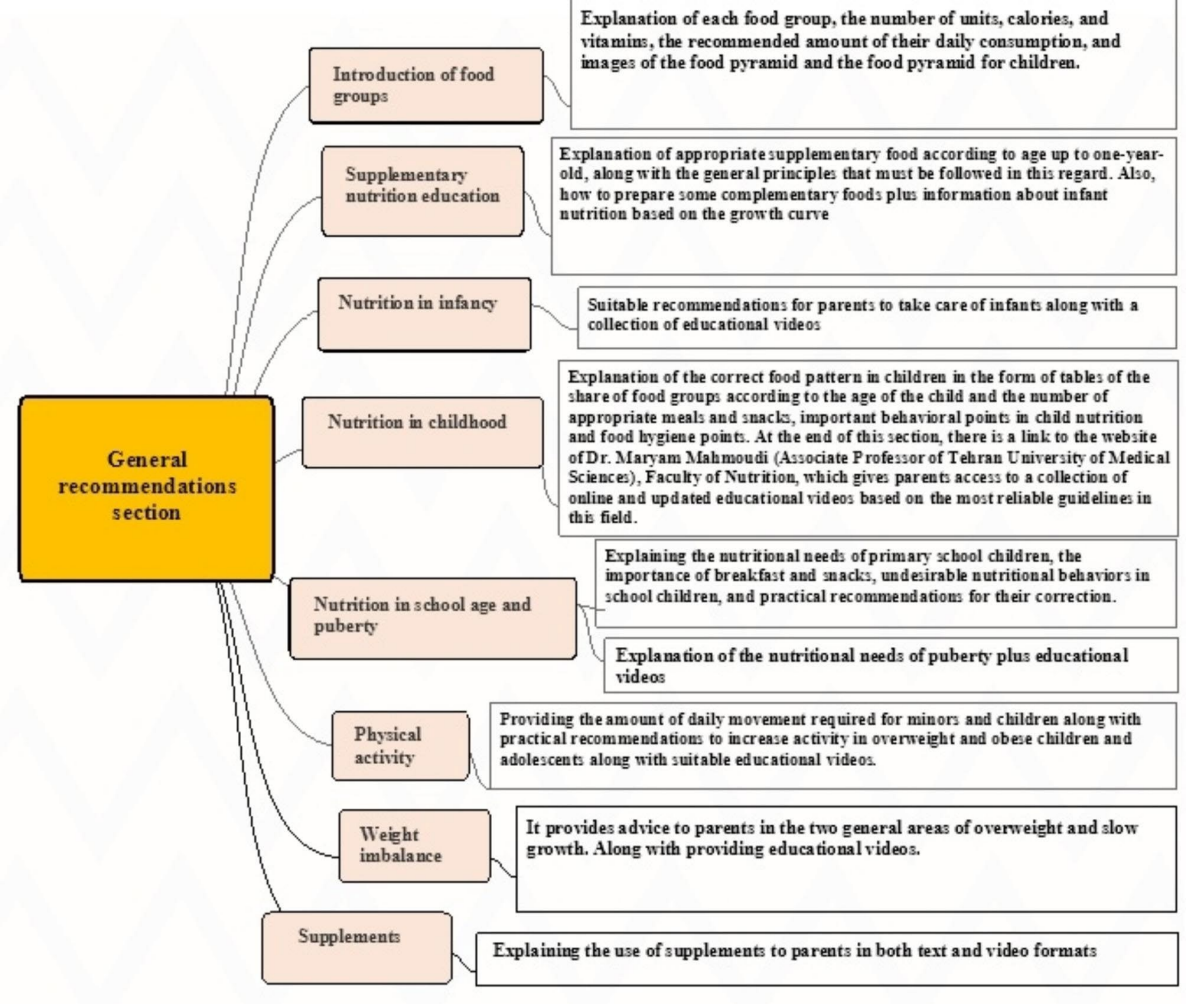
General recommendations

This section has eight parts, introducing food groups, education on supplementary nutrition, nutrition in infancy, nutrition in childhood, nutrition in school age and puberty, physical activity, weight imbalance, and supplements (Fig. 6).

In all the above cases, to see the specific training videos, it is necessary to refer to the “videos” option, which is embedded on the top left of the page.

### The section of charts’ trends over time

Among the important features of this app, we can refer to the “charts’ trend” section, which is smart and displays the charts’ trends according to the child’s age. This section easily provides parents with the progress of their children’s development. In fact, in the registration section, parents enter the child’s date of birth and gender only once, and later in time the charts and their trends are calculated and displayed separately according to the child’s age and gender.

If the child is under two years of age, the saved records can be seen in the two sections of “weight chart trend according to age” and “height chart trend according to age”. Each of these two charts provides the child’s growth curve, which is obtained by connecting the marked points in each growth measurement. Therefore, in this section, parents can access their children’s “electronic growth card” that is automatically prepared by the software. Each of these trends has explanations on how to interpret the curve.

If the child is over 2 years old, the related trends section will also contain BMI percentile charts according to age, which can be seen based on the date of growth measurement, and in this sense, it appears different from the trends section related to the age group of under 2 years.

### The section of child psychology assessment

**Fig. 7:**
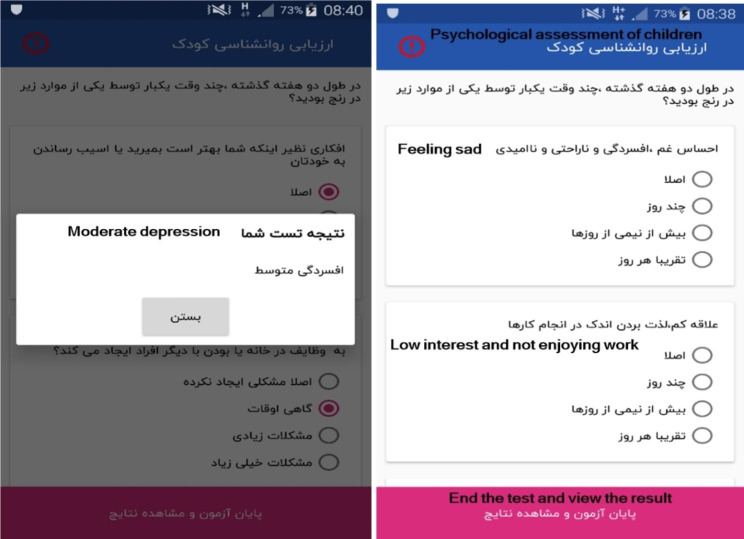
Pages related to psychology assessment

This section has a set of questions that evaluate children under the age of 18 in terms of depression. On this page (Fig. 7), a “warning” icon can be seen at the top of the left page, which explains that this test is used for the initial assessment of the child’s psychological status and is not a basis for a definitive diagnosis. If the result of this test is positive, the parents will be recommended to refer the child to a relevant specialist for specialized assessment (in order to diagnose depression or anxiety).

### Reminders

**Fig. 8:**
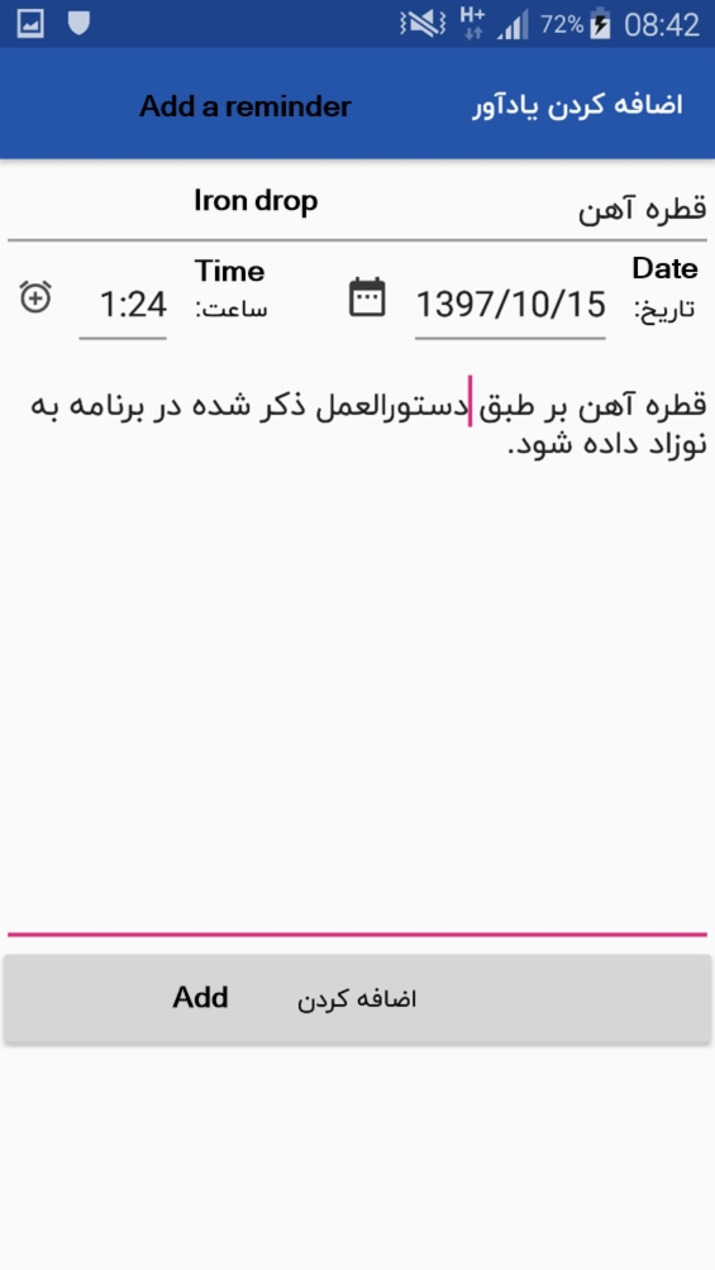
Reminders

The last section allows parents to record the date, time, and text of the reminder in order to remind them about items, such as supplementary food, snack, vaccination, etc. (Fig. 8).

**Fig. 9:**
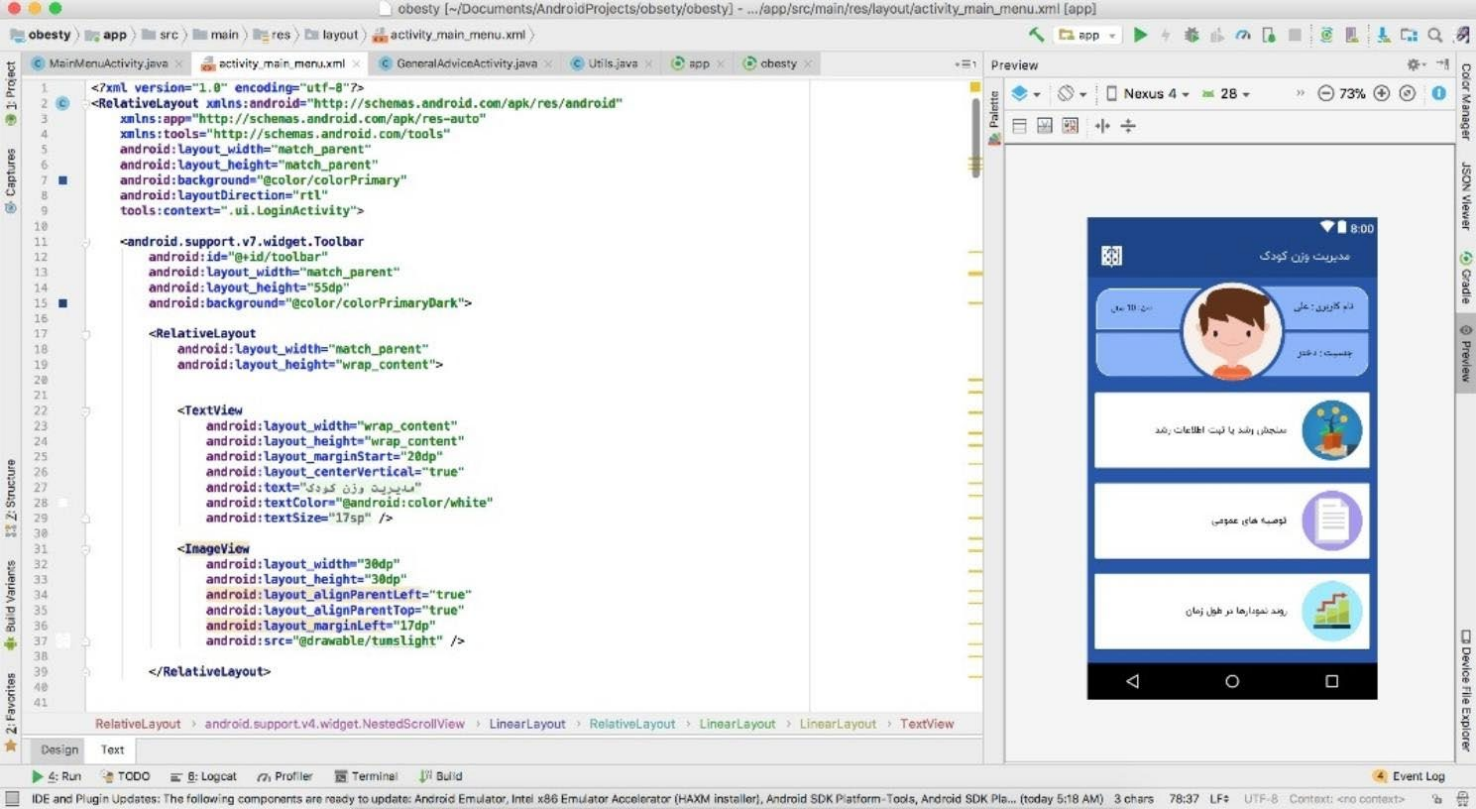
Codes related to the main page of the app

An example of the codes written in the Android Studio 3 programming environment is shown in Fig. 9.

### Usability evaluation of the app

To evaluate the usability of this app, a total of 20 participants, including nutritionists and regular users, were recruited. The individual variables considered in this evaluation included age, gender, and level of education. The frequency of male participants in the evaluation phase of the program (n = 7, 35%) was lower than female participants (n = 13, 65%). The level of education of the participants included 3 undergraduates, 2 graduates, 5 bachelors, 1 master’s degree, and 9 nutritionists.

The QUIS questionnaire included five parts of the overall performance of the program, screen capabilities, set of terms and information of the program, ability to learn the program and general capabilities of the program.

The analysis of data obtained from the usability evaluation and user satisfaction of the smartphone-based app to prevent and control overweight and obesity in children are shown in the table below (Table 1). The first part of the questionnaire related to questions about overall performance has an average users score of 8.45, which is at the “good” level. The second part of the questionnaire was related to the app’s screen capabilities, which has an average score of 8.3, which was evaluated at the “good” level. The third part of the questionnaire was related to the terminology and information section, which has an average score of 7.55, which was evaluated at the “good” level. The fourth part of the questionnaire was related to learning capabilities, which has an average score of 7.98, which was evaluated at the “good” level. Finally, the fifth part of the questionnaire was related to general capabilities, which has an average score of 7.59, which is evaluated at the “good” level.

**Table 1:** The result of usability evaluation and user satisfaction with the app

No	Users’ opinions about	Mean	Standard Deviation (SD)	Allocation of mean in classes (6.1 to 9)	Classification of scores
1	Overall performance of the application	8.45	0.75	۞	Good
2	Screen capabilities	8.3	1.32	۞	Good
3	A set of terms and information of the program	7.55	1.24	۞	Good
4	Learnability of the program	7.98	2.02	۞	Good
5	General features of the program	7.59	1.22	۞	Good

## Discussion

In this study, a smartphone-based app was designed and evaluated to control and prevent overweight and obesity in children. The various versions of this app were designed in the Android Studio 3 programming environment and using the Java 8 programming language. This app can be used on phones with the Android operating system. At the suggestion of users, more educational information was added regarding supplementary nutrition and nutritional diets, as well as supplements. Some users wanted to simplify and explain the evaluation results as much as possible in terms of growth tables, and additional explanations were considered in this regard and included in the program. The performance of the app, the ease of access, and the use of different parts of the app were measured through QUIS. The results of the evaluation and the average scores obtained in the questionnaire show the applicability of the app at a favourable level. This level of acceptance of the software by users can be attributed to the presence of parents and experts in the software design stages and receiving feedback from them and applying comments on the software to improve it. User-centered design led to the identification of changes needed to improve the ease of use of the app and made it easy for users to use.

Numerous studies have found that smartphone technology is able to change the traditional way of face-to-face education and provide a new method of education. Also, in terms of time and place, this technology provides learners with flexible learning time and places even at home and the workplace. It has also removed many limitations and inefficiencies in this regard. Telephone communication, as the most basic form of electronic health plan, can be an important and valuable aspect of patient care plans [34].

On the other hand, studies have shown that satisfactory adherence to treatment and, as a result, weight loss or maintenance can be achieved through the use of smartphone applications [35]. In fact, these applications, by tracking calorie consumption and physical activities, enable users to lose weight better than traditional methods [19]. Therefore, such applications can be used to establish appropriate interaction between parents and overweight or obese children, and also provide timely and effective guidance to them.

In this app, users can find educational information such as “correct measurement of height and weight”, as well as diagrams and charts necessary to interpret the growth rate of children according to their age group and gender. This feature reduces the probability of error in choosing the correct chart by parents, helping them to be easily aware of their children’s nutritional status by regularly monitoring their growth. In fact, monitoring the child’s growth is one of the best and most effective ways of preventing malnutrition in childhood [36]. It can also serve as an early warning for parents, allowing them to take an appropriate and timely intervention, and also identify any problem in the child’s growth and development before it turns into malnutrition. Moncho and colleagues state that interventions aimed at achieving healthy eating habits and patterns will have a positive effect on the health of future children and adults. Therefore, the goal of public health policies and interventions should be the prevention and treatment of childhood obesity. Also, by eliminating social and economic inequalities, the health system should provide adequate support to at-risk groups [37].

In this app, the existence of charts and instruction on how to use them will provide children and parents with appropriate feedback on the weight management process, which can be a suitable and important incentive for them. Studies have shown that behavioral variables, such as self-monitoring and appropriate feedback, are the key components of weight management interventions and their optimization can lead to more efficient weight management in children and adults [38].

In the general recommendations section, users can have access to special information such as food groups, complementary nutrition, nutrition in infancy, nutrition in childhood, nutrition in school age and puberty, physical activity, weight imbalance and supplements, and in all the above cases, they can use special educational videos for further training. In their study, Woźniak and colleagues showed that providing appropriate education on nutrition to parents can be effective in improving the nutritional status of children at the social level [39]. The study of Zoghby and colleagues has also shown that better communication between parents and physicians and, as a result, higher awareness of parents about childhood obesity is associated with a better attitude and performance of parents towards childhood obesity [40].

Another section that parents have at their disposal in this app is the section of child psychology assessment, which examines the child in terms of depression. In this test, if the result is positive, the parents will be recommended to refer the child to the relevant specialist for specialized assessment. In this regard, many studies have shown that children and adolescents who are being treated for obesity have a higher risk of developing emotional-psychological problems and these problems are positively correlated to their level of obesity [41]. Although not all obese children experience psychosocial problems, some are at risk of associated psychiatric problems. Therefore, it is necessary to assess children in terms of psychosocial problems, because social stigma that exists in relation to obesity has the potential to significantly affect the motivation and consequently the treatment of obese children [42].

In the last section of the designed app, parents have the possibility to record the date, time and text of reminder to remind them about items, such as use of supplementary food, vaccination or the use of necessary supplements, etc. Such measures can increase the child’s immunity and health [43]. In this regard, Yunusa and colleagues showed that smartphone reminders were effective in completing the vaccination course [44].

The results of the usability evaluation and user satisfaction questionnaire showed that these two features of the designed app have been evaluated at a favorable level by the users. However, Ghelani and colleagues in their study revealed that the time-consuming and relatively difficult input of dietary data on smartphone applications received a less enthusiastic response from the participants [38]. In this context, it can be inferred that the advantages of the designed application include its user-friendly interface and convenient accessibility. The high degree of user acceptability may be attributed to the involvement of parents and specialists throughout the various stages of software development. Furthermore, the application’s implementation of user-centered design facilitated the discernment of necessary modifications aimed at enhancing its usability.

In 2016, Con et al. [45] conducted a systematic review of content and tools, examining applications based on Android and iOS operating systems available in the mobile market that were designed for the self-management of inflammatory bowel diseases. Their findings were based on the fact that among all the applications identified in the Android and iPhone mobile markets, 69% of the applications were based on the Android operating system, which is in line with the operating system used in the present study.

In Mahdizadeh’s study entitled “Design and construction of a remote skin disease diagnosis system” in 2015, the researcher also used the think-aloud method and user satisfaction questionnaire to evaluate the system’s usability after creating the prototype. They asked five people (three physicians and two patients) to use all features of the system and express their opinions. Then, the requested changes were applied in the system, and after completing the system, its final version was used to evaluate its usability and user satisfaction. The results of the data analysis showed that the system was evaluated at a good level by the users [46].

The usability and user satisfaction evaluation of designed systems in other studies are in line with the present research [26, 27, 47]. In general, the results obtained from the evaluation and opinion polling of users showed that, this app can help parents to measure their child’s development and monitor their nutritional status without paying unnecessary visits to medical centers.

### Strengths and limitations

One of the strengths of the present study is that the design of the app is based on the opinions of experts and scientific texts. Also, the app was evaluated in two separate rounds and the main users were also included in the evaluation phase. After detecting serious problems and the suggestions of experts, the app was developed in its last version. It should be noted that, despite the capabilities of the designed app, it also has some limitations. For instance, this app has only been designed to operate on the Android operating system. Therefore, the results of this study are limited to this system and cannot be generalized to other systems. It should be noted that the sample size of users in this study is limited, consisting exclusively of Android users who possess a high level of education. Consequently, the findings may not accurately represent the perspectives of individuals with lower technological literacy or education levels, who may be more vulnerable to obesity. One further constraint pertains to the potential discrepancy between the usability assessment conducted by experts and users in limited time and the real usage and benefits experienced by users. Consequently, more investigation is needed to comprehensively analyse the app’s impact.

## Conclusion

Overweight and obesity are among the most important health problems in the world, including in our country. This problem is increasing all over the world at an alarming rate and has many negative effects on the quality of life of people in childhood and adulthood. Early diagnosis of obesity can help prevent or treat it because it’s crucial to change one’s lifestyle in order to improve one’s quality of life and, as a result, control and manage children’s weight. Smartphones with features such as the ability to download and install self-management programs have played a significant role in disease control, diagnosis and management, as well as patient education. In fact, the potential of e-health can be used to support the self-management of chronic patients and, as a result, improve the health status and quality of life of patients, reduce healthcare costs and achieve patient satisfaction. A smartphone weight management app for children and adolescents was designed and evaluated in this study. By using this app, people can become familiar with the causes and symptoms of weight imbalance and manage their weight as best as possible. They can also use this app to monitor and improve their nutritional status and that of their children.

## Data Availability

All data generated or analyzed during this study are included in this published article.

## References

[1] *World Health Organization. Obesity and Overweight: Key facts*. accessed on 24 April 2022.

[2] Agha M, Agha R (2017). The rising prevalence of obesity: part A: impact on public health. Int J Surg Oncol.

[3] Lister NB (2023). Child and adolescent obesity. Nat Reviews Disease Primers.

[4] Calcaterra V (2023). Sugar-Sweetened Beverages and metabolic risk in children and adolescents with obesity: a narrative review. Nutrients.

[5] Vaisi-Raygani A (2019). The prevalence of obesity in older adults in Iran: a systematic review and meta-analysis. BMC Geriatr.

[6] Brzeziński M (2020). Lipid disorders in children living with overweight and obesity-large cohort study from Poland. Lipids Health Dis.

[7] Rumgay H (2022). Global, regional and national burden of primary liver cancer by subtype. Eur J Cancer.

[8] Rashidi A (2005). Prevalence of obesity in Iran. Obes Rev.

[9] Sanyaolu A et al. *Childhood and adolescent obesity in the United States: a Public Health concern*. Glob Pediatr Health, 2019. 1(6). 10.1177/2333794X19891305 PMC6887808 31832491

[10] akbari n (2005). Comparative survey of parents’BMI in obese and non-obese. - ‎12‎ year old Children in Esfahan, 2000 Journal of Holistic Nursing and Midwifery.

[11] Rathnayake KM, Roopasingam T, Wickramasighe VP (2014). Nutritional and behavioral determinants of adolescent obesity: a case–control study in Sri Lanka. BMC Public Health.

[12] Anderson Y, Chew CSE. *Consequences of Childhood and Adolescent Obesity*, in *Clinical Obesity in Adults and Children*. 2022. p. 339–352.

[13] Roberts KJ, Polfuss ML. *Weight stigma in children and adolescents: Recommendations for practice and policy* 2022. 52(6): p. 17–24. 10.1097/01.NURSE.0000829904.57766.58 35609070

[14] Nasim M (2019). Identifying obesity/overweight status in children and adolescents; a cross-sectional medical record review of physicians’ weight screening practice in outpatient clinics, Saudi Arabia. PLoS ONE.

[15] Doupis J (2020). Smartphone-based technology in Diabetes Management. Diabetes Therapy: Research Treatment and Education of Diabetes and Related Disorders.

[16] Barbosa HC (2021). Empowerment-oriented strategies to identify behavior change in patients with chronic diseases: an integrative review of the literature. Patient Educ Couns.

[17] Bao Y (2022). Effects of an mHealth intervention for pulmonary tuberculosis self-management based on the integrated theory of health behavior change: randomized controlled trial. JMIR Public Health and Surveillance.

[18] Feroz A, Jabeen R, Saleem S (2020). Using mobile phones to improve community health workers performance in low-and-middle-income countries. BMC Public Health.

[19] Haleem A (2021). Telemedicine for healthcare: capabilities, features, barriers, and applications. Sens Int.

[20] Azzopardi-Muscat N, Sørensen K (2019). Towards an equitable digital public health era: promoting equity through a health literacy perspective. Eur J Pub Health.

[21] Shahmoradi L (2016). Comparing three data mining methods to predict kidney transplant survival. Acta Informatica Medica.

[22] Melzner J, Heinze J, Fritsch TJPT (2014). Mob Health Appl Workplace Health Promotion: Integr Concept Adoption Framew.

[23] Grange ES et al. *Responding to COVID-19: the UW medicine information technology services experience* 2020. 11(02): p. 265–275. 10.1055/s-0040-1709715 PMC7141898 32268390

[24] Patterson V. J.F.i.p.h., *Telemedicine for epilepsy support in resource-poor settings* 2014. 2: p. 120. 10.3389/fpubh.2014.00120 PMC4139740 25191650

[25] Leigh JW (2022). Smartphone ownership and interest in mobile health technologies for self-care among patients with chronic heart failure: cross-sectional survey study. JMIR Cardio.

[26] Shahmoradi L (2021). Prevention and control of urinary tract stones using a smartphone-based self-care application: design and evaluation. BMC Med Inf Decis Mak.

[27] Shahmoradi L (2022). Predicting the survival of kidney transplantation: design and evaluation of a smartphone-based application. BMC Nephrol.

[28] Chávez A (2019). Design and evaluation of a mobile application for monitoring patients with Alzheimer’s disease: a day center case study. Int J Med Informatics.

[29] O’Grady C (2020). A mobile health approach for improving outcomes in suicide prevention (SafePlan). J Med Internet Res.

[30] Lee EY, Yoon K-H (2018). Epidemic obesity in children and adolescents: risk factors and prevention. Front Med.

[31] Hajizadeh E (2021). Identification of the minimum data set to design a mobile-based application on overweight and obesity management for children and adolescents. J Diabetes Metab Disord.

[32] Asghari Amrei S, Ayatollahi H, Salehi SH (2020). A smartphone application for burn self-care. J Burn Care Res.

[33] Chin JP, Diehl VA, Norman KL. *Development of an instrument measuring user satisfaction of the human-computer interface*. in *Proceedings of the SIGCHI conference on Human factors in computing systems*. 1988.

[34] Goodarzi M, Ebrahimzadeh I. *Impact of Distance Education via short message service of Mobile Phone on metabolic control of Patients with Type 2 Diabetes Mellitus in Karaj-Iran. %J Internal Medicine Today* 2014. 19(4): p. 224–234.

[35] Dounavi K, Tsoumani O (2019). Mobile Health Applications in Weight Management: a systematic literature review. Am J Prev Med.

[36] Ulasi TO et al. *Knowledge and perception of growth monitoring among caregivers attending a tertiary level* Health Care Facility 2021. 11(5).

[37] Moncho J, Martínez-García A, Trescastro-López EM. *Prevalence of overweight and obesity in children of immigrant origin in Spain: a cross-sectional study*. 2022. 19(3): p. 1711. 10.3390/ijerph19031711 PMC8834947 35162734

[38] Ghelani DP (2020). Mobile apps for Weight Management: a review of the latest evidence to inform practice. Front Endocrinol.

[39] *The Influence of Parents’ Nutritional Education Program on Their Infants’Metabolic Health* 2022. 14(13): p. 2671. 10.3390/nu14132671 PMC9268789 35807852

[40] Zoghby HB (2022). Knowledge, attitude and practice of lebanese parents towards childhood overweight/obesity: the role of parent-physician communication. BMC Pediatr.

[41] Pan L (2017). Psychological assessment of children and adolescents with obesity. J Int Med Res.

[42] Sagar R, Gupta T (2018). Psychological aspects of obesity in children and adolescents. Indian J Pediatr.

[43] Yunusa U (2021). Mobile phone reminders for enhancing uptake, completeness and timeliness of routine childhood immunization in low and middle income countries: a systematic review and meta-analysis. Vaccine.

[44] Yunusa U (2022). Effect of mobile phone text message and call reminders in the completeness of pentavalent vaccines in Kano state, Nigeria. J Pediatr Nurs.

[45] Con D, De Cruz P (2016). Mobile phone apps for inflammatory bowel disease self-management: a systematic assessment of content and tools. JMIR mHealth and uHealth.

[46] Mehdizadeh H (2015). Designing and Building a Teledermatology System %J. J Mazandaran Univ Med Sci.

[47] Amiri P (2023). A Mobile Application to assist Alzheimer’s caregivers during COVID-19 pandemic: development and evaluation. J Caring Sci.

